# An Evaluation of the Effects of the Transobturator Tape Procedure on Sexual Satisfaction in Women with Stress Urinary Incontinence Using the Libido Scoring System

**DOI:** 10.1155/2013/627671

**Published:** 2013-10-28

**Authors:** Raziye Narin, Hakan Nazik, Mehmet Ali Narin, Hakan Aytan, Murat Api

**Affiliations:** ^1^Department of Obstetrics and Gynecology, Adana Numune Education and Research Hospital, 01330 Adana, Turkey; ^2^Department of Obstetrics and Gynecology, University of Mersin, Mersin, Turkey; ^3^Department of Obstetrics and Gynecology, Zeynep Kamil Education and Research Hospital, Istanbul, Turkey

## Abstract

*Introduction and Hypothesis*. Most women experience automatic urine leakage in their lifetimes. SUI is the most common type in women. Suburethral slings have become a standard surgical procedure for the treatment of stress urinary incontinence when conservative therapy failed. The treatment of stress urinary incontinence by suburethral sling may improve body image by reducing urinary leakage and may improve sexual satisfaction. *Methods*. A total of 59 sexually active patients were included in the study and underwent a TOT outside-in procedure. The LSS was applied in all patients by self-completion of questionnaires preoperatively and 6 months after the operation. General pleasure with the operation was measured by visual analogue score (VAS). Pre- and postoperative scores were recorded and analyzed using SPSS 11.5. *Results*. Two parameters of the LSS, orgasm and who starts the sexual activity, increased at a statistically significant rate. *Conclusion*. Sexual satisfaction and desire have partially improved after the TOT procedure.

## 1. Introduction

Urinary incontinence can be described as an involuntary urine loss, which has become a social or hygienic problem. The prevalence of urinary incontinence varies widely and increases with age [1]. Most women experience automatic urine leakage in their lifetimes. SUI is the most common type in women [2]. In this condition, increase of intra-abdominal pressure causes urine leakage during coughing, laughing, or weightlifting. SUI can be demonstrated objectively by some tests including stress test and Q-tip test. The transobturator tape procedure was developed by Delorme to reduce the complications associated with the tension-free vaginal tape (TVT) including bladder perforation, urinary retention, and bowel injury [3]. Recently, suburethral slings have become a standard surgical procedure for the treatment of stress urinary incontinence when conservative therapy failed.

Libido is a subjective issue that refers to a person's sex drive or desire for sexual activity. The definition and explanation of this term has been problematic since the symptoms are vague and can be seen in other conditions too, for example, depressive disorders. Moreover, no cutoff level for a normal range of libido has been agreed on [4].

The treatment of stress urinary incontinence by a suburethral sling procedure may improve body image by reducing urinary leakage. Thus, a positive effect on sexual satisfaction could be expected. Conversely, sexual satisfaction may worsen by developing dyspareunia after the suburethral sling procedure [5].

In this study, we aimed to evaluate the effects of the TOT procedure on female sexual satisfaction using LSS and determine the level of pleasure with the operation.

## 2. Materials and Methods

This prospective observational study was performed at the Department of Gynecology of Numune Education and Research Hospital in Adana, Turkey. The inclusion criteria for the study were women younger than 65 who had documented SUI symptoms, a positive stress test, and a sexually active life. All of the postmenopausal women were taking hormone replacement therapy (HRT). The exclusion criteria included pregnancy, a previous pelvic surgery, severe complaints of urgency incontinence, and urinary tract infection not responding to therapy. A total of 59 sexually active women who underwent solely the transobturator tape outside-in procedure (I-stop, mesh, polypropylene) between January 2012 and October 2012 were included in this study. The patients were discharged when the postvoid residual volume was less than 100 mL. Their sexual satisfactions were scored using self-administered questionnaire libido scoring system preoperatively and 6 months postoperatively. The libido scoring system (LSS) was developed in 1997 by Api et al. and involves four questions on four domains: orgasmic function, coital frequency, sexual desire, and sexual self-interest (masturbation) (Table 1) [6]. The (Female Sexual Function Index) FSFI was well-correlated with the LSS, revealing a correlation coefficient of 0.96 (*P* < 0.001) (Cronbach *α* coefficient was found to be 0.83 and the kappa values of total scores were 0.67 and 0.77) [6].

The patients were scored according to Table 1 and a total score less than 3 was considered as loss of libido; scores of 3 and 4 were considered as low libido, 5–7 as moderate libido, and 8–12 as high libido.

The patients were asked to ask themselves four questions to fill out the self-completed questionnaire:

1. how often do you have sex or masturbate?
2. do you masturbate?
3. who starts the sexual activity? (Who asks for or implies sex first)
4. do you orgasm by yourself and/or with your partner?

Their general satisfaction with the TOT procedure was evaluated with a visual analogue score (VAS). This gives the greatest freedom and maximum opportunity for each respondent to express their personal pleasure. Such VAS data are recorded in millimeters from the left of the line with the range from 0 to 100. All patients were asked to put a mark on the line wherever they wanted.

All women were asked to give informed consent. The study was approved by the medical ethics committee of the Numune Hospital.

Statistical analysis was carried out on a personal computer using the Statistical Package for Social Sciences version 11.5 (SPSS 11.5, demo, SPSS Inc., Chicago, IL). Kolmogorov-Smirnov test with the Lilliefors correction was used to test whether the variables used in the study were normally distributed. Normally distributed data were expressed as mean ± standard deviation, and data that were not normally distributed were expressed as medians (interquartile range). Wilcoxon's signed-ranks test was used to compare the differences between preoperative and postoperative parameters. Spearman's rho correlation coefficients were used for correlation analysis purposes. The statistical significance level was set at 5%.

## 3. Results

Of all women who underwent a TOT procedure alone during the study period, 59 were eligible and agreed to take part in the study.

The mean age was 45.1 ± 9.5 years, and the mean (Body Mass Index) BMI was 28.8 ± 3.5 kg/m^2^. The median values for gravidity, parity and VAS scores of patients with interquartile ranges were 4 (3), 3 (1), and 85 mm (34), respectively.

Tables 2 and 3 show the previous surgeries that the patients underwent and the accompanied diseases they had.

Table 4 shows the changing of incontinence, sex frequency, who starts the sexual activity, orgasm and total libido scores preoperatively and 6 months postoperatively, and *P* values.

Masturbation frequency did not change in any patient. The rate of patients who masturbated pre- and postoperatively was 37.3% (*N* = 22).

As shown in Table 4, all parameters (incontinence, who starts the sexual activity, orgasm, and total score), with the exception of sexual intercourse frequency, improved significantly when compared to the preoperative period.

**Figure 1:**
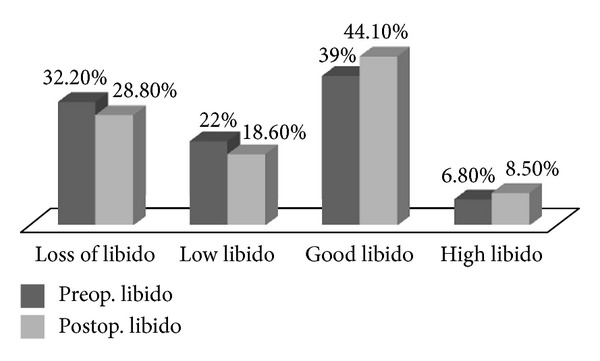
It shows changing libido before and after sling operation.

As shown in Figure 1, loss of libido and low libido rates decreased (from 32% to 28% and from 22% to 18.6%, resp.), and moderate and high libido rates increased (from 39% to 44% and from 6.8% to 8.5%) after the transobturator tape procedure. There was no correlation between the VAS and the difference between the pre- and postprocedure total libido (Spearman's Rho: 0.128, *P* = 0.334).

## 4. Discussion

In this study, prospective data have been presented on the effects of the TOT procedure on sexual satisfaction in women. Satisfaction with the sexual life seems to be partially improved after the TOT procedure.

It is common knowledge that libido is a subjective matter in men and women involving several aspects including desire, pleasure, sexual life, intercourse, erection, ejaculation, orgasm, and happiness clitoral or penile sensation [6]. Temml et al. reported that 25% of women had impairment of their sexual life by urinary incontinence [7]. Uncontrolled urination and smell of urine may cause embarrassment and reduce self-confidence. This mood causes some sexual disorders including loss of focus on sexual intercourse, avoidance, hesitation, and fear of humiliation. All such negative emotions may reduce sexual desire and satisfaction. The women's self-esteem can be strengthened by greater urinary control. We designed this research based on the idea that sexual life will improve as a result. A total of 59 patients were included in this study, and their pre- and postsurgery libidos were compared. Besides, overall pleasure with the surgery was measured by VAS. Our findings demonstrate that two parameters of the libido scoring system (who starts the sexual activity and orgasm), total libido scores, and continence ratios get better after the sling procedure. Increase in orgasm and starting the sexual activity may indicate increased enjoyment of sexual intercourse. The rate of masturbation remained the same. The frequency of sexual intercourse improved in 15 patients but was statistically insignificant. There was no correlation between general pleasure with the TOT procedure and changing of total libido scores. Women who took part in the present study were followed up for 6 months postoperatively. A longer follow-up period could indicate the sexual satisfaction change over time.

For the TOT procedure, Sentilhes et al. [5] reported 31% improvement and 10% deterioration in intercourse satisfaction. Similarly, in our study, the libido improved by 40.7%, whereas the deterioration rate was 15.3%. Typically, incontinence cure rate after the transobturator tape procedure is very high. In our study, the total subjective cure rate was 81.4%.

In a systematic review and meta-analysis by Jha [8] about the impact of incontinence surgery on sexual function on eighteen studies which analyzed 1578 women, the combined analysis of all studies showed that, in just over half of all women, there was no change of overall sexual symptoms after surgery (55.5%). For midurethral tapes alone, there was no significant improvement of sexual function. Our data showed that two parameters (who starts the sexual activity and orgasm) improved at a statistically significant level by the TOT procedure. This may be explained by increasing body image and self-esteem just by reducing stress urinary incontinence. Similarly, Arts-de Jong et al. reported a significant improvement of satisfaction with sexual function after incontinence surgery [9].

Sexuality can be measured through patient-doctor interviews as well as through self-completion questionnaires. It is important that such questionnaires should be simple and easy for the patient to complete [10]. Moreover, questionnaires must be applicable to the target population based on their cultural, educational, ethnic, and religious backgrounds [11, 12]. According to the same meta-analysis [8], questionnaires used in studies included the Prolapse and Incontinence Sexual Function Questionnaire (PISQ 31 and 12), Female Sexual Function Index (FSFI) [13], Lemack, Bristol Female Lower Urinary Tract Symptoms questionnaire (BFLUTS), and the electronic Pelvic Floor Assessment Questionnaire (ePAQ). The libido scoring system (LSS) was not used earlier to evaluate sexual satisfaction following sling procedures. The correlation between FSFI, the mostwidely used diagnostic test, and LSS was shown [6]. For this reason, we preferred a simpler test LSS instead of FSFI. 

There are some limitations of this study. Firstly, our study group was small. The other weakness is a single-point evaluation at the 6-month follow-up. Collecting patient data at additional intervals of 12 months might have made the study stronger. 

## 5. Conclusion

The TOT procedure partially improves female sexual satisfaction. This could be via reducing urinary incontinence and raising self-esteem. Extended observation periods may elucidate the changes in sexual satisfaction over time.

## Figures and Tables

**Table 1:** Total libido scoring.

Item/score	0	1	2	3
Sexual frequency	None	≤once a week	Twice a week	>twice a week
Masturbation	Does not			Does
Who starts the sexual activity	Always partner	Mostly partner	Mostly herself/himself	Always herself/himself
Orgasm	Never	Sometimes	Frequently	Always

Total scoring:

0–2 scores: loss of libido.

3-4 scores: low libido.

5–7 scores: good libido.

8–12 scores: high libido.

**Table 2:** Previous surgeries of patients.

	Frequency % (*N*)
C/S	5.1 (3)
L/S tubal ligation	5.1 (3)
Appendectomy	6.8 (4)
Thyroidectomy	3.4 (2)
TAH + C/S + BTL	1.7 (1)
TAH	3.4 (2)
C/S + BTL	1.7 (2)

Total	27.1 (16)

C/S: cesarean section, L/S: laparoscopy, TAH: total abdominal hysterectomy, BTL: bilateral tubal ligation.

**Table 3:** Accompanied diseases.

	Frequency % (*N*)
HT	8.5 (5)
HT + DM	11.9 (7)
Asthma	5.1 (3)
DM	5.1 (3)
Behçet	1.7 (1)

Total	32.2 (19)

HT: hypertension, DM: diabetes mellitus.

**Table 4:** Variation of parameters before and after surgery.

	Improvement % (*N*)	No change % (*N*)	Deterioration % (*N*)	P
Incontinence	81.1 (48)	15.2 (9)	3.4 (2)	<0.001*
Sex frequency	24.4 (15)	62.8 (37)	11.8 (7)	0.27
Who starts the sexual activity	24.4 (15)	66.1 (39)	8.5 (5)	0.02*
Orgasm	27.1 (16)	67.8 (40)	5.1 (3)	0.01*

Total score	40.7 (24)	44.0 (26)	15.3 (9)	0.01*

*P ≤ 0.05 statistically significant. Wilcoxon signed-ranks test.
